# Fast detection and quantification of *Plasmodium* species infected erythrocytes in a non-endemic region by using the Sysmex XN-31 analyzer

**DOI:** 10.1186/s12936-022-04147-0

**Published:** 2022-04-11

**Authors:** Tania A. Khartabil, Yolanda B. de Rijke, Rob Koelewijn, Jaap J. van Hellemond, Henk Russcher

**Affiliations:** 1 Department of Clinical Chemistry, Erasmus MC University Medical Center, Rotterdam, The Netherlands; 2 Department of Medical Microbiology and Infectious Diseases, Erasmus MC University Medical Center, Rotterdam, The Netherlands

**Keywords:** Malaria, Diagnosis, Flow cytometry, Hemocytometry, *Plasmodium*

## Abstract

**Background:**

Due to increased travel from endemic countries, malaria occurs more frequently in non-endemic regions. It is a challenge for diagnostic laboratories in non-endemic countries to provide reliable results, as experience of staff is often limited to only a few cases per year. This study evaluated the diagnostic accuracy of the fully automated Sysmex XN-31 malaria analyzer in a routine diagnostic setting in a non-endemic region was evaluated.

**Methods:**

Samples from 112 patients suspected for malaria were examined by the Sysmex XN-31 analyzer to determine the absolute count of malaria-infected red blood cells count (MI-RBC/µL). Microscopic examination of both Quantitative Buffy Coat capillary tubes and thick and thin blood films were used as reference methods. Limits of blank (LoB), detection (LoD) and quantification (LoQ) were investigated using an in vitro *Plasmodium falciparum* culture. Nine hundred twenty samples of patients with RBC abnormalities were included to determine which RBC abnormalities trigger indeterminate or false positive results.

**Results:**

No false positive nor false negative results were obtained for the examined patient samples suspected for malaria. For 3% of samples an indeterminate result by the XN-31 was obtained. The Passing-Bablok regression line for diagnostic accuracy of the parasitaemia was y = 39.75 + 0.7892 × showing a positive bias of about 21% when comparing the MI-RBC results to microscopy. The LoB, LoD and LoQ were calculated to be 4.7, 5.9, and 19.0 infected RBC/μL, respectively. From the 920 abnormal RBC samples collected, 4.6% resulted in a false positive MI-RBC result and almost half of the samples produced indeterminate results. These results were related to increases in nucleated red blood cells, reticulocytes and other abnormal RBC morphologies such as sickle cells.

**Conclusions:**

Based on the results, the XN-31 is a fast and reliable screening method in the detection and quantification of *Plasmodium* species in patients However, if an abnormal red blood cell morphology is present, the results of the XN-31 should be interpreted with caution as false positive results can be caused by interfering abnormal erythrocytes.

## Background

Malaria is a life-threatening disease caused by the protozoan parasite *Plasmodium,* which is transmitted through the bite of an infected *Anopheles* mosquito. In 2019 there were 229 million infections with 409,000 deaths reported globally [1]. Most of these cases occur in sub-Saharan Africa, which is responsible for 93% of cases and 94% of deaths [1]. Due to the increasing number of global travellers and immigration from endemic countries malaria becomes more relevant in non-endemic countries in moderate climates as well. The five common *Plasmodium* species known to infect humans are *Plasmodium* *falciparum*, *Plasmodium vivax*, *Plasmodium ovale*, *Plasmodium malariae* and *Plasmodium knowlesi*. More than 90% of malaria cases and the most severe and possibly fatal disease are caused by *P. falciparum* [2].

Early and accurate diagnosis of malaria is fundamental for successful and timely treatment of the disease, as delay and/or misdiagnosis can result in morbidity and mortality. According to the World Health Organization (WHO), it is recommended to have prompt malaria diagnosis either by microscopy or by a malaria rapid diagnostic test (RDT) in all patients with suspected malaria before treatment is administered [3]. Microscopic examination of thick and thin blood films remains the golden standard according to current CDC guidelines and approximately 10–25 µL of blood is used to make both blood films [4]. This method, however, is time consuming and requires the availability of an experienced microscopist to examine the blood films, which is often a challenge outside office hours and significant variability in applied methods exist among laboratories [5]. Therefore, RDTs for malaria have been developed of which immunochromatographic card tests (ICT) that detect antigens of *Plasmodium* in blood of the host, are most commonly used. These ICTs are easy to perform, use approximately 10–20 µL of blood, and provide results within 10–15 min [6]. However, in case of infections with a low parasitaemia, false negative results can be obtained [7]. Furthermore, the sensitivity of RDT testing is decreasing for the detection of *P. falciparum* due to specific mutations in, or complete deletion of, the *hrp-2* gene of *P. falciparum* [8]. Alternative methods like Loop Mediated Isothermal Amplification (LAMP), Real-Time PCR (rt-PCR) and Quantitative Buffy Coat (QBC) fluorescence analysis can be used as reliable alternative screening methods, but are either time consuming, expensive and/or in need of trained staff to be available 24 h a day, seven days a week (24/7) [5, 9–11]. Hence, no ideal laboratory method is currently available that provides fast and reliable results with information on both the *Plasmodium* species (as these can require different therapy) and the parasitaemia value needed to assess the disease severity of patients infected with *P. falciparum* or *P. knowlesi* without the need of well-trained technicians. This is especially relevant for diagnostic laboratories in non-endemic countries, because these encounter often only a few malaria cases per year and thus lack experience to diagnose malaria by microscopic examination of blood films [5].

The Sysmex XN-31 haemocytometer is an automated analyzer launched in September 2019 to support malaria diagnosis in whole blood samples in the clinical diagnostic laboratory. Using fluorescence flow cytometry (FFC) technology and a violet semiconductor laser with a 405 nm wavelength, this haemocytometer can detect, specify and quantitate malaria-infected red blood cells (MI-RBC) within a specific area of the scattergram known as the M-gating area. Previous studies on the XN-31 and its predecessor the XN-30 reported mainly data on *P. falciparum* infected patients in endemic countries [12–16] or on in vitro cultures [13, 17, 18]*.* The XN-30, was approved for research purposes only, whereas the XN-31 is a CE marked in vitro diagnostic device with identical hardware, software, and user interface. The XN-31 has not been evaluated in clinical practice in non-endemic countries nor for *Plasmodium* species other than *P. falciparum* and *P. vivax* [13, 14, 16].

In this study, the performance of the XN-31 in clinical practice was compared to the current diagnostic workflow at Erasmus Medical Center, which is based on ICT in combination with microscopy by QBC analysis and thin and thick blood film examination. In addition, the limit of blank (LoB), limit of detection (LoD), and the limit of quantification (LoQ) were determined and compared to previous values reported by Sysmex in the Instructions for Use. Since the XN-31 can report indeterminate results that have been suggested to be linked to interference caused by certain RBC abnormalities, such as are commonly observed in peripheral blood films of patients with sickle cell disease or other haemoglobinopathies [12]. The specificity of *Plasmodium* infected RBC detection by the XN-31 was investigated by examining 920 blood samples derived from patients with a wide array of RBC abnormalities.

## Methods

### Sample inclusion

One hundred and twelve EDTA whole blood samples, for which malaria examination was ordered by the physicians, were collected at the Erasmus MC University Medical Center in Rotterdam, the Netherlands, between December 2019 and December 2020, including the follow-up samples of patients after initiation of treatment. In addition, 32 samples from asymptomatic, healthy individuals with no suspicion of malaria were collected for the determination of the Limit of Blank (LoB). Samples that were older than 24 h post collection or had a volume of less than 500 μL were excluded. To investigate which RBC abnormalities, perturb the MI-RBC examination, blood samples were selected from routine haematology of patients that were not suspected of malaria, comprised a high number of NRBC (> 5%) and a relatively normal WBC count of < 20 (× 10^3^/µL). NRBC was used as the primary selection criterion as these frequently co-exist with other RBC abnormalities. With these selection criteria, 920 blood samples were examined with a high amount of NRBCs, reticulocytes and/or morphological RBC abnormalities from patients with thalassaemia, sickle cell disease without haemato-oncological diseases, and ICU patients with infections and stress erythropoiesis.

### Malaria examination reference methods

At Erasmus Medical Center the standard procedure to diagnose malaria in freshly collected EDTA-blood specimens involves an ICT RDT for malaria antigens and microscopic examination of both a QBC capillary and stained thick and thin blood films. The rapid diagnostic antigen test (Binax NOW® Malaria Test Binax, Inc. Maine, USA) and the QBC analyses were performed according to the manufacturer’s instructions. QBC capillaries were examined independently by two technicians by microscopic analysis of two complete rows of the region between the bottom of the capillary and the polynuclear leukocyte layer using an Olympus BX-60 fluorescence microscope equipped with UV-filter, 50× objective and 12.5× oculars (total magnification 625×). Thick blood films were stained with Field’s stain (Waldeck GMBH & CO KG, Münster, Germany) and thin blood films were fixed with absolute methanol for three minutes and subsequently stained with Diff Quick (RAL Diagnostics, Martillac, France). Both staining procedures had been optimized for optimal staining of *Plasmodium* parasites as well as Schüffner’s dots and Maurer’s clefts in infected erythrocytes. Thick and thin films were examined with regular light microscopes at a total magnification of 1,250x. For all *Plasmodium* positive blood specimens the *Plasmodium* species was confirmed by a real-time PCR method based on the method of Shokoples et al. [19].

### Flow cytometry analysis

The XN-31 was operated in the Low Malaria (LM) mode, as this mode uses a three times higher sample volume (60 µL) compared to other modes, which lowers the detection limit and thus increases the sensitivity of the method. Using this Low Malaria mode of analysis, the XN-31 provides a complete blood count (CBC), a qualitative result (positive, negative or indeterminate for malaria-infected red blood cells, MI-RBC), a quantitative result (an absolute MI-RBC count and the percentage of *Plasmodium* infected RBC) and a result for the suspected *Plasmodium* species (*P. falciparum*, or *Plasmodium* non-falciparum, or *Plasmodium* sp.). Speciation provided by XN-31 is a suspect flag approved for research use only purposes by the manufacturer. All study samples were also processed on the Sysmex XN-1000 Series analyzer to collect full profile data (complete blood count, CBC), white blood cell differential (WDF), and reticulocytes (RET). The XN-31 results for malaria were compared to those of the combined results of RDT, QBC and rt-PCR analyses in order to determine the negative predictive value (NPV), positive predictive value (PPV), and efficiency. The XN-31 MI-RBC count includes all malaria-infected RBCs, irrespective of life stage. The differentiation into sexual (gametocytes) and non-sexual (rings, mature trophozoites, schizonts) are research use only parameters. The quantification of infected erythrocytes on the XN-31 for both sexual and asexual stages of *Plasmodium* parasites were compared to the counting results obtained by microscopic examination of thin and thick blood films.

The LoB assessment was based on 32 malaria-negative blood samples from healthy individuals with no symptoms and no suspicion for malaria. These samples had CBC values on the Sysmex XN-1000 hemocytometry analyzer within the reference ranges used at the Department of Clinical Chemistry at Erasmus Medical Center. The LoB was calculated using the following formula: LoB = mean blank + 1.645(1SD of blank sample).

The LoD was determined using in vitro cultured RBC infected with *P. falciparum* NF54 parasites (a generous gift of Dr. M. McCall, Radboudumc, Nijmegen, The Netherlands), that were serially diluted in freshly collected full blood of a healthy donor. The LoD was calculated by the following formula; LoD = LoB + 1.645 (1SD of the sample with the lowest MI-RBC concentration above the LoB with a reproducible qualitative test result).

The LoQ was calculated from the same dilution series and based on the point in the dilution series that exceeded a coefficient of variation (CV) of 20%. Quantification of infected RBC was also determined by microscopic examination of thick and thin blood films. Thin blood films were used to count infected erythrocytes if the parasitaemia was above 4783 parasites per µL and thick blood films were used to quantify parasites for all dilutions with a lower parasitaemia.

### RBC abnormality interferences on XN-31

In addition to a positive or negative result for MI-RBC, the XN-31 can also provide an indeterminate result. Blood films were made for all these XN-31 examined samples to confirm the presence of RBC morphologies. The XN-31 scattergrams of all these samples were compared to true positive *Plasmodium* samples in order to investigate the potential causing interference. The selected blood samples were examined by the XN-31 haemocytometer and thin blood films were prepared and examined to confirm the presence of RBC morphologies. The XN-31 scattergrams of all these samples were compared to true positive *Plasmodium* samples in order to investigate the potential causing interference.

### Statistical analysis

Data analysis was performed by using Analyse-it for Microsoft Excel version 2.30 and Microsoft Excel 2016. Passing-Bablok regression analysis was used to determine accuracy of the MI-RBC produced by the XN-31 compared to microscopy.

## Results

### Performance of the XN-31 in clinical practice

Due to the COVID-19 pandemic and the drop in international travellers during the time of this study, the number of requests for malaria examination was substantially decreased compared to the pre-COVID-19 period. There were 112 included and 14 of them contained *Plasmodium* parasites based on the results of analysis by RDT, QBC, thin and thick blood film examination and rt-PCR. These 14 positive samples were derived from 8 patients, because 6 samples were follow-up samples after initiation of malaria treatment. The 8 malaria patients were infected with *P. falciparum* (n = 6)*, P. malariae* (n = *1)* or *P. vivax* (n = 1). Of the 112 samples, 109 were correctly diagnosed by the XN-31, either as negative (n = 96) or positive (n = 13) and for those the *Plasmodium* species were correctly determined as *P. falciparum* or *Plasmodium* non-falciparum. The XN-31 produced an indeterminate result for the remaining three samples and, therefore, the XN-31 had a positive and negative predictive value of 100% within an efficiency of 96% (Table 1). Of the three samples for which an indeterminate result was reported, one sample contained *P. malariae*. The parasitaemia in this sample appeared to be very low as only sporadically infected erythrocytes were found in thick and thin blood films. In addition, this sample was also determined to contain microfilaria of *Mansonella perstans*. The other two samples that had an indeterminate XN-31 result did not contain *Plasmodium* infected RBC.

**Table 1:** Performance of XN-31 compared to the combination of parasitological examinations used in routine patient care

Overall result of parasitological examinations		
	Positive	Negative
XN-31 result		
Positive	13	0
Indeterminate	1	2
Negative	0	96
Total	14	98

Predictive value and efficiency		
PPV	NPV	Efficiency
100%	100%	96%

PPV: positive predictive value, defines the probability of having *Plasmodium* in a sample with a positive result. NPV: negative predictive value, describes the probability of not having *Plasmodium* in a sample with a negative test result. Efficiency is the proportion of correctly classified samples as negative or positive among all samples

**Fig. 1:**
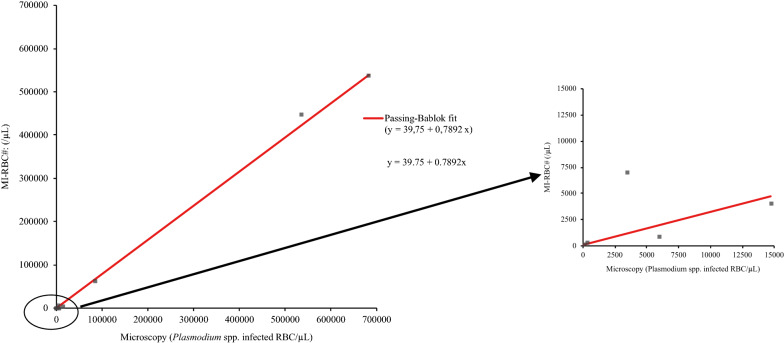
Accuracy of XN-31 parasitaemia determination in clinical patient samples compared to parasitaemia determined by microscopy

**Fig. 2:**
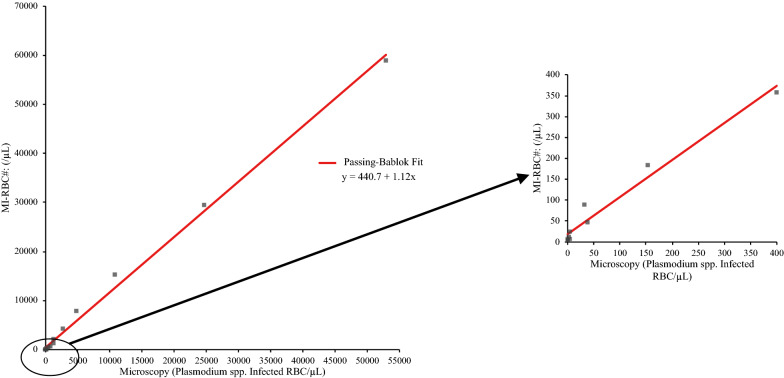
Accuracy of XN-31 for dilution series of in vitro cultured *P. falciparum* compared to microscopy. A linear dilution series was prepared of in vitro cultured *P. falciparum* infected RBC in freshly obtained blood of a healthy donor, after which the parasitaemia was determined by XN-31 and microscopic examination of blood films. Line of best fit is in red comparing the results of the XN-31 MI-RBC# (/μL) to the microscopy results

To investigate the correlation between the parasitaemia determination by the XN-31 and microscopic examination, Passing-Bablok analysis was performed on 12 of the 14 positive samples. For one positive sample the parasitaemia could not reliably be determined by microscopy because the patient was treated for malaria long enough that the parasite morphology in infected erythrocytes was too aberrant to be reliably determined. In addition, in the *P. malariae* sample too few infected erythrocytes were present that the parasitaemia could not be accurately be determined by microscopy. On the 12 remaining positive samples, Passing-Bablok analysis was performed with the parasitaemia results from the XN-31 and the parasitaemia determined in thin and thick blood films to determine the accuracy of the XN-31 across a range of distinct concentrations of *Plasmodium* infected RBC. The parasitaemia of the XN-31 correlated well with the parasitaemia determined by microscopy (Fig. 1) with a proportional bias of 21% (y-intercept of 39.75 and slope of 0.79). In addition, quantification of *Plasmodium* infected RBC in a dilution series prepared from in vitro cultured RBC infected with *P. falciparum* in freshly collected blood of a healthy donor, demonstrated a good agreement between the parasitaemia determined by the XN-31 and microscopic methods as well as a clear linear response with a best fit line of y = 348.1 + 1.13x (Fig. 2).

### Determination of XN-31 detection limits for *Plasmodium* infected erythrocytes

**Fig. 3:**
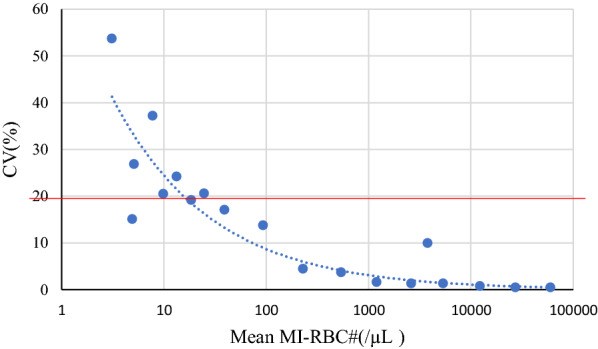
Determination of Limit of Quantification (LoQ) of the XN-31. Each point in the graph represents the mean of 10 replicates in that concentration and the %CV associated with those replicates. The LoQ is the lowest concentration of infected erythrocytes with a %CV < 20 (indicated with the red line), which is in this case an MI-RBC of 19 parasites/μL. LoQ: limit of quantification; CV: coefficient of variation

As shown in Table 2 the LoB was determined to be 4.7 infected RBC/μL and the LoD was determined to be 5.9 infected RBC/μL. In addition, a dilution series of in vitro cultured *P. falciparum* was used to determine the LoQ. As shown in Fig. 3 the lowest concentration at which the %CV was still below 20% was 19/μL, which had a CV of 19%.

**Table 2:** Determination of LoB and LoD for the XN-31

Determination of LoB for MI-RBC# (/μL)	
Mean #MI-RBC of blanks (32 samples)	2.4
1SD	1.4
LoB = mean blank + 1.645(1SD of blank sample)	4.7

Determination of LoD for MI-RBC# (/μL)	
Mean #MI-RBC of Target LoD Sample (10 replicates)	4.9
1SD	0.7
LoD = LoB + 1.645 (1SD of low conc. sample)	5.9

LoB was calculated using samples of patients not infected with *P. falciparum*. The LoD was calculated based on the LoB and 1SD of the dilution sample mean with a reproducible positive test result with the lowest number of MI-RBCs above the LoB. LoB: limit of blank; LoD: limit of detection; SD: standard deviation

### RBC abnormalities interfering with XN-31 analysis

In order to examine which RBC abnormalities could trigger indeterminate results, 920 samples from 254 unique patients with RBC abnormalities (ranging from 1 to 16 samples per patient) were selected from regular patient care haemocytometry. Included samples contained no *Plasmodium* parasites, had greater than 5% NRBC’s and less than 20 × 10^9^ WBC/L, or came from the haematology clinic. Processing of these 920 blood samples on the XN-31 generated 449 negative, 429 indeterminate and 42 false positive results for the detection of *Plasmodium* infected RBC. The MI-RBC values of these false positive samples varied substantially as a wide range of 20 to 44,310 infected RBC/μL was observed. This result demonstrates that the false positive results did not only occur with low MI-RBC values being incorrectly detected. Table 3 shows the frequency of occurrence of specific RBC abnormalities (as determined by the XN-1000) in samples classified by XN-31 as positive, negative or indeterminate for MI-RBC qualitative judgment. Upon reviewing the results for these samples on the XN-1000, more than 67% of the samples with an MI-RBC false positive result and 62% of the samples with indeterminate result had greater than 10% NRBCs. It is clear in Table 3 that there are lower percentages of samples with increased NRBCs and reticulocytes that are triggering the false positive MI_RBC results. There were also samples with lower numbers of NRBC and reticulocytes triggered an indeterminate or false positive MI-RBC result. Therefore, thin blood films of all false positive MI-RBC samples, were manually re-evaluated by two trained microscopists to examine the abnormal RBC morphology present in these samples. In total 42 samples from 31 patients gave false positive MI-RBC results. Of these, 15/42 samples (36%) were from 8 sickle cell disease patients with sickle cells present in their blood films. In the examined group of abnormal RBC samples especially the sickle cell patients triggered many a false positive or an indeterminate MI-RBC result and never a negative result apart from the few samples from sickle cell patients for which no sickle cells in their blood films could be found. Furthermore, false positive or indeterminate MI-RBC results were also generated with the samples from premature newborns (6/42), having high numbers of reticulocytes and NRBC counts. Other patient groups and RBC anomalies occurring in the false positive MI-RBC result group were haemochromatosis (8/42), beta thalassaemia (12/42), leukaemia/lymphoma (6/42), and sepsis (1/42). Many patients in the false positive MI-RBC group had multiple diagnoses and had multiple samples taken at different time points. Many patients in the false positive MI-RBC group had multiple diagnoses, notable all haemochromatosis samples came from a single patient with beta thalassaemia.

**Table 3:** Detection of *Plasmodium* infected RBCs on the XN-31 for routine blood samples with RBC abnormalities based on XN-1000 results

Sysmex XN-1000 Parameter (# out of 920)	XN-31 Result for the detection of *Plasmodium* infected RBCs		
	Negative (% out of 920)	Indeterminate (% out of 920)	Positive (% out of 920)
NRBC > 10% (404)	111 (12%)	265 (29%)	28 (3%)
RET > 1.5% (699)	251 (27%)	411 (45%)	37 (4%)
RBC ABN Flag (448)	105 (11%)	313 (34%)	30 (3%)
RET ABN SCAT (251)	22 (2%)	205 (34%)	24 (3%)
Total	489	1194	119

Most samples included are comprised of more than a single abnormality

NRBC: nucleated red blood cell; RET: reticulocytes; WBC ABN: white blood cell abnormal; RBC ABN: red blood cell abnormal; RET ABN SCAT:reticulocyte abnormal scattergram

**Fig. 4:**
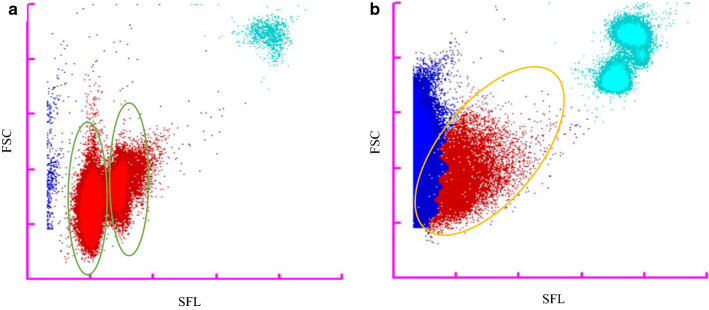
Comparison of XN-31 scattergrams of a true positive *Plasmodium falciparum* sample with a false-positive MI-RBC sample. **A** Scattergram of a true positive MI-RBC samples containing erythrocytes infected with *Plasmodium falciparum*. **B** Scattergram of a sample of a patient in sickle crisis that produced a false positive MI-RBC result by the XN-31. The red particles are what the XN-31 suspects to be a *Plasmodium* infected red blood cell*,* the teal particles are the leukocytes, and the dark blue particles are the non-infected red blood cells or debris. In **A**, a true positive sample, the cluster of events is vertical (green circles), whereas the false-positive events identified in the MI-RBC channel in the sickle cell crises samples cluster at a 45° angle (orange circle in panel B). FSC: forward scatter light; SFL: side-fluorescent

To determine whether a true positive MI-RBC sample can be distinguished from a false positive MI-RBC sample, we compared the MI-RBC scattergrams of the XN-31 analyses. In Fig. 4, a representative example of a true positive MI-RBC sample and a false positive sample are shown. The events in the forward scatter light and side-fluorescent light (FSC and SFL) scattergram of a true positive MI-RBC sample demonstrate a compact and perpendicular pattern with the defined clusters of RBC infected by one or multiple ring forms as explained by Pillay et al. [12] (shown in the green circles in Fig. 4A). On the other-hand the scattergrams of false positive MI-RBC samples demonstrate a dispersed pattern at a 45 °C angle (located within the orange circle) (Fig. 4B).

## Discussion

The results of this study show that the XN-31 can be used in clinical practice as a fast and easy screening assay for malaria that provides reliable qualitative and quantitative results. Therefore, XN-31 can easily be integrated in regular 24/7 patient care diagnostics settings in non-endemic counties and can provide all required information to clinicians to timely start proper treatment. Compared to other screening assays, the XN-31 is the only test that can provide rapid results (< 1 min) to determine the presence of *Plasmodium-*infected RBC with a detection limit equivalent to thick blood film examination [5] in combination with a *Plasmodium* species differentiation and an accurate parasitaemia quantification. The XN-31 can accurately determine the parasitaemia in samples with low numbers of infected erythrocytes, as the limit of detection and quantification was determined to be 5.9 and 19 infected RBC per µL, respectively. Thereby the detection limit of the XN-31 is equivalent to thick and thin blood examination which has on average a detection limit of ~ 10 parasites per µL [5].

Although there were no false positive or false negative results for samples of patient suspected for malaria in this study, it is known that submicroscopic malaria exists [20]. In these cases the number of infected erythrocytes is below the detection limit of thick blood film examination, and thus also below the detection limit of the XN-31. Therefore, false negative results can occur, but most submicroscopic malaria cases are asymptomatic and occur in patients from endemic areas with extensive immunity against malaria or in patients infected with a benign, non-falciparum, *Plasmodium* species that will present with a typical and characteristic fever pattern returning every 48 or 72 h [21–23]. Hence, for patients whom malaria is clinically suspected, but a negative result is obtained, regardless of which test is used, repeat testing should be undertaken periodically. In specific cases further examinations by more sensitive methods can be indicated for patients for which a negative result by the XN-31 has been generated. In this analytical performance evaluation an LoD of ~ 6 MI-RBC/µL was achieved, which is significantly lower than the 20 MI-RBC/µL cut-off set for qualitative judgment of MI-RBC present or absent. The threshold for defining a sample as positive could therefore possibly be adjusted by the manufacturer.

Next to the hypothetical possibility of false negative results due to patients with very low parasitaemia, this study demonstrated that indeterminate and false positive MI-RBC results can occur as well. Examination of a large panel of blood samples of patients with a variety of RBC abnormalities demonstrated not only a high frequency of indeterminate results (~ 50%), but also false positive results (~ 5%). These false positive MI-RBC results were predominantly encountered for blood samples from patients with sickle cell disease (HbSS and HbSβ0) and patients with increased numbers of nucleated erythrocytes and/or reticulocytes. Conditions of stressed erythropoiesis such as may occur in thalassaemia or other haemoglobinopathies are mentioned in the instructions for use as potentially giving an erroneous MI-RBC positive result. Analysis of the scattergrams demonstrated substantial differences between the true positive and false positive MI-RBC samples and, therefore, future refinement of the automatic interpretation script of the XN-31 for the obtained scattergrams should result in improved performance of the XN-31. Thus, caution is required for patients with abnormal blood cell morphology and review of the scattergram is advised when authorizing results. It should however be noted that in this study the number of samples measured was deliberately enriched with those expected to cause interferences and that the actual occurrence of such issues may be substantially lower in the routine setting where only samples from patients suspected to have malaria will be measured on XN-31.

In order to better determine what could produce indeterminate and false positive MI-RBC results by the XN-31, abnormal RBC samples were examined. For detection of MI-RBC, the software determines the number of events in the M-gating area as shown in Fig. 4. If the number of detected events exceeds a certain threshold an indeterminate or positive result will be generated according to clustering patterns defined by algorithms. When interfering cell types are present that produce a scattergram with a distinct cluster of particles in the M-gating area, the algorithm will override the presence of generalized background scatter, producing a false positive MI-RBC result. This also means that when 20 or more parasites/μL are present, but no distinct cluster can be detected due to interference, an indeterminate result will be produced that is marked with an abnormal scattergram flag. Examination of the panel of abnormal RBC samples showed that a number of diseases and conditions frequently occurred in both the false positive and indeterminate MI-RBC results group; beta thalassaemia major, leukaemia, lymphoma, premature newborns, and sickle cell disease. The observation occurring most frequently for the false positive samples was sickle cell disease in crisis. Although there were no sickle cell patients included in the study population for suspected *Plasmodium* infection, it can be hypothesized that a patient with sickle cell disease and malaria should get a valid MI-RBC present result (cluster detected) although the actual parasitaemia value would be overestimated. Premature newborns, beta thalassaemia and haemochromatosis are associated with stressed or disturbed erythropoiesis as well and we speculate that triggering of indeterminate and false positive results is highly correlated with diseases and conditions associated with the presence of immature cells in the erythrocyte lineage or with severely abnormal RBC morphology.

Although in this study the population of patients suspected for a *Plasmodium* infection, none of the mentioned diseases were present, it is very well possible as RBC abnormalities occur relatively frequently in the population in malaria endemic areas [24]. Some of these abnormalities can even cause mortality in malaria patients making it even more important to understand exactly which RBC abnormalities cause indeterminate or positive result on the XN-31. Mortality in sickle cell patients with malaria is a problem in endemic countries that have a high prevalence of sickle cell disease. More than 80% of people that have sickle cell disease live in sub-Saharan Africa where most *Plasmodium* deaths occur [25]. In one study from Cameroon, it was found that 2 out of every 10 sickle cell patients who died had malaria [26]. To date, there have been preliminary studies done how interferences affect the results of the XN-31, but there is some discrepancy in the results and what conditions can trigger an abnormal scattergram on the XN-31 [14, 15]. For the investigation of RBC abnormalities, abnormal samples were deliberately collected to determine the effect on the XN-31 result. This does not necessarily represent the frequency of false positive results in endemic regions where thalassaemia and sickle cell disease are more prevalent as there have been studies performed previously on the XN-30 in regions such as Burkina Faso that showed no false positive results [16]. However, it is important to augment this data with further studies in order to determine whether the XN-31 can properly detect *Plasmodium* infected RBC in patients with sickle cell disease and other diseases that significantly affect red blood cell morphology and/or erythropoiesis.

When an indeterminate result occurs, it is clear that additional examinations by other methods are required to confirm whether *Plasmodium* infected RBC are present or not to prevent the reporting of a false positive MI-RBC result, which could lead to misdiagnosis. To mitigate the possibility of having a false positive MI-RBC result with potentially serious consequences, it is recommended to evaluate the haemocytometric scattergrams in order to determine whether or not there is distinct cluster formation of particles within the M-gating area where parasites are detected, as seen in a true positive *Plasmodium* sample. In case of abnormal RBC morphology and a positive malaria result, *Plasmodium* infected RBC should be confirmed by microscopic examination of thick and/or thin blood films. However, the scattergrams of true positive and false positive MI-RBC samples are different and therefore further refinement of XN-31 gating and interpretation algorithms should be able to increase the specificity.

Finally, there are some limitations of this study. Firstly, the number of included patients for diagnostic accuracy was smaller than anticipated due to travel restrictions during the COVID-19 outbreak. Secondly, the LoD and LoQ studies were performed using *P. falciparum* parasites cultured in vitro in RBC as there were no patient samples with a parasitaemia high enough to dilute serially across the linear range. Although the use of in vitro cultures on the XN-31 is not approved in the specifications, the results show no interferences were present if the dilution series is prepared by dilution of *P. falciparum* parasites cultured in RBC in vitro in freshly collected blood of a healthy donor. Using this method accurate results were obtained for the LoB, LoD, and LoQ.

## Conclusion

The XN-31 is a promising alternative for rapid diagnostic antigen tests, as mutations in the *hrp-2* gene will not interfere with the accuracy of results on the XN-31. However, this study demonstrated that false positive results can occur in sickle cell patients or other RBC abnormalities such as elevated NRBCs and/or reticulocytes and confirmation by the reference method is necessary. However, the scattergrams of true positive and false positive MI-RBC samples are different and therefore further refinement of XN-31 gating and interpretation algorithms should be able to increase the specificity. And until that has been achieved, caution is required for patients with abnormal blood cell morphology. Ultimately, this study shows that the XN-31 can be a fast and accurate screening method for the detection and quantification of *Plasmodium* infected RBC in blood samples of patients suspected for malaria.

## Data Availability

The data that support the findings of this study are available from Erasmus MC, Department of Clinical Chemistry but restrictions apply to the availability of these data, which were used under license for the current study, and so are not publicly available. Data are however available from the authors upon reasonable request and with permission of Erasmus MC, Department of Clinical Chemistry.
